# Optimising the timing of renal replacement therapy in acute kidney injury

**DOI:** 10.1186/s13054-021-03614-5

**Published:** 2021-05-31

**Authors:** Matthew E. Cove, Graeme MacLaren, Daniel Brodie, John A. Kellum

**Affiliations:** 1 Department of Medicine, National University Singapore, NUHS Tower Block, Level 10, 1E Kent Ridge Road, Singapore, 119228 Singapore; 2 Cardiothoracic ICU, National University Hospital, 5 Lower Kent Ridge Road, Singapore, 119074 Singapore; 3 Columbia University College of Physicians and Surgeons/New York-Presbyterian Hospital, New York, NY USA; 4 Center for Acute Respiratory Failure, New York-Presbyterian Hospital, New York, NY USA; 5 Department of Critical Care Medicine, Center for Critical Care Nephrology, University of Pittsburgh, 606 Scaife Hall, 3550 Terrace Street, Pittsburgh, PA 15261 USA

**Keywords:** Continuous renal replacement therapy, Intensive care, Acute kidney injury

## Abstract

The optimal timing of renal replacement therapy (RRT) in critically ill patients with acute kidney injury (AKI) has been much debated. Over the past five years several studies have provided new guidance for evidence-based decision-making. High-quality evidence now supports an approach of expectant management in critically ill patients with AKI, where RRT may be deferred up to 72 h unless a life-threatening indication develops. Nevertheless, physicians’ judgment still plays a central role in identifying appropriate patients for expectant management.

## Introduction

Initiation of organ support therapy is a complex decision integral to practice in the intensive care unit (ICU). Such decisions extend beyond whether these therapies simply normalise relevant physiological parameters and include triage, prognostication and resource allocation [1, 2], as well as an assessment of the potential for the intervention to cause more harm than benefit in individual patients. When considering renal replacement therapy (RRT) for acute kidney injury (AKI), critical care physicians are required to balance the hazards of starting too early, risking patient exposure to an unnecessary therapy with its attendant complications and costs [3, 4], against the potential life-threatening harm of initiating too late [4, 5].

In certain circumstances, decision making is relatively clear. There is little doubt RRT should be started promptly in the presence of life-threatening indications (hyperkalaemia, severe metabolic acidosis, pulmonary oedema, certain drug toxicities), and it is equally clear that RRT is not necessary when these indications are absent and there is evidence of recovery from AKI. RRT is also inappropriate when it is incompatible with patient preferences and treatment goals. However, many critically ill patients continue to meet AKI criteria without developing either an indication for urgent dialysis or evidence of imminent recovery. For these patients, clinicians face a difficult question. Should RRT be initiated early, risking over-treatment, or should it be delayed as long as possible, potentially prolonging exposure to fluid overload and solute imbalance? Five important studies over the past 5 years have attempted to address this question [6–10].

The first two of these studies were published in 2016; the ‘early versus delayed initiation of renal replacement therapy on mortality in critically ill patients with AKI’ (ELAIN) trial [6] and the ‘initiation strategies for renal-replacement therapy in the intensive care unit’ study from the ‘artificial kidney initiation in kidney injury’ (AKIKI) group [7]. The ELAIN trial was a single centre study recruiting 231 ICU patients with stage 2 AKI, based on the Kidney Disease: Improving Global Outcomes (KDIGO) guidelines (Table 1) [11]. To be eligible, patients needed at least one modifier of illness severity (severe sepsis, vasopressor use, fluid overload or progression of other organ dysfunction) and an elevated plasma neutrophil gelatinase-associated lipocalin (NGAL) (Table 2). Patients were randomised to receive early RRT within 8 h, or late therapy starting more than 12 h after the patient had reached KDIGO stage 3 AKI criteria. Mortality at 90 days was lower in the early group (39.3% vs 54.7%, *p* = 0.03), and all-cause mortality remained significantly lower at 1 year (50.2% early vs 69.8% delayed; *p* = 0.01) [12].

**Table 1:** Definitions of AKI

Kidney disease: improving global outcomes (KDIGO) guideline [7]			Acute dialysis quality initiative (ADQI) group [8]		
Staging	Serum creatinine criteria	Urine output criteria	Staging	Serum creatinine criteria	Urine output criteria
1	1.5–1.9 times baseline OR Increase ≥ 0.3 mg/dL	< 0.5 ml/kg/h for 6–12 h	Risk	Increased × 1.5	< 0.5 ml/kg/h for 6 h
2	2.0–2.9 times reference value	< 0.5 ml/kg/h for ≧12 h	Injury	Increased × 2	< 0.5 ml/kg/h for ≧12 h
3	> 3.0 times baseline OR Increase to ≧4 mg/dL OR Initiation of renal replacement therapy	< 0.3 ml/kg/h for ≧24 h	Failure	Increased × 3 OR Increase to ≧4 mg/dL (Acute rise ≧0.5 mg/dL)	< 0.3 ml/kg/h for 24 h OR Anuria for 12 h

**Table 2:** Comparison of key renal RRT studies in critically Ill patients with AKI

Study	Design (year)	Sample Size	Entry criteria	Groups	Outcome
Early versus delayed initiation of RRT on mortality in critically ill patients with AKI (ELAIN) [6]	Single centre RCT (2016)	231	KDIGO stage 2 AKI, NGAL > 150 ng/mL, ≥ 1 of: severe sepsis, vasopressor use, fluid overload or progression of other organ dysfunction	Early, RRT started within 8 h OR Late, RRT started 12 h after developing Stage 3 AKI*	90 Day Mortality. Early = 39.3%, Late = 57.4% (*p* = 0.03)
Artificial kidney initiation in kidney injury (AKIKI) study group [7]	Multicentre RCT (2016)	620	KDIGO Stage 3 AKI and mechanical ventilation or catecholamine infusion or both	Early, RRT started within 6 h OR Late, RRT started if oliguria persisted > 72 h*	60 Day Mortality. Early = 48.5%, Late = 49.7% (*p* = 0.79)
Initiation of dialysis early versus delayed in the intensive care unit (IDEAL-ICU) study [8]	Multicentre RCT (2018)	488	Septic shock and meeting RIFLE ‘F’ criteria	Early, RRT started within 12 h OR Late, RRT started after 48 h	90 Day Mortality. Early = 58%, Late = 54% (*p* = 0.38)
Standard versus accelerated initiation of RRT in AKI (STARRT-AKI) trial [9]	International Multicentre RCT (2020)	2927	KDIGO Stage 2 or 3 AKI	Accelerated RRT, within 12 h OR Standard, RRT started after 72 h*	90 Day Mortality, Accelerated = 43.9%, Standard = 43.7% (*p* = 0.92)
Comparison of two delayed strategies for RRT initiation for severe AKI (AKIKI 2): a multicentre, open-label, randomised, controlled trial [10]	Multicentre RCT (2021)	278	KDIGO Stage 3 AKI and mechanical ventilation or catecholamine infusion or both. Oliguria or anuria for > 72 h or BUN 112 – 140 mg/dL	Delayed group, RRT start in < 12 h. More delayed group, RRT postponed until BUN ≥ 140 mg/dL or urgent indication	RRT-free days Delayed = 12 days More delayed = 10 days HR^#^ for death 60 days 1.65 (95% CI 1.09–2.50) for more delayed group

^*^RRT started earlier if urgent indication developed such as, metabolic acidosis, hyperkalemia or hypoxemia attributable to fluid overload. ^#^HR = Hazard ratio 1.65 (95% CI 1.09–2.50, *p* = 0.018)

The AKIKI study group appeared to report conflicting findings. These investigators conducted a multicentre trial in 31 intensive care units (ICUs) and recruited 620 patients with KDIGO AKI stage 3 who required either mechanical ventilation, use of vasopressors, or both [7] (Table 2). Patients were randomised to an early group receiving RRT immediately, or a delayed group, where therapy was only started if a life-threatening indication developed, blood urea nitrogen increased to 112 mg/dL (urea 40 mmol/L), or oliguria persisted for 72 h. The primary outcome, 60 day mortality, was comparable in both groups (48.5% early vs 49.7% late, *p* = 0.79).

Since the publication of ELAIN and AKIKI, three subsequent studies have been completed, with designs and outcomes similar to the AKIKI trial (Table 2). In 2018, the ‘initiation of dialysis early versus delayed in the intensive care unit’ (IDEAL-ICU) study randomised 448 patients with septic shock and severe kidney injury to receive RRT within 12 h of documented renal failure (using Risk, Injury, Failure, Loss of kidney function, and End-stage kidney disease or RIFLE classification [13] Table 1), or delayed support after 48 h if renal recovery had not occurred [8]. The RIFLE criteria for renal failure used in this study were comparable to the criteria used for KDIGO AKI stage 3 (Table 1). This multicentre trial was conducted in 29 ICUs and found no significant difference in 90 day mortality between early or delayed RRT (58% early vs 54% delayed RRT, *p* = 0.38).

In 2020, the larger ‘standard versus accelerated initiation of RRT in AKI’ (STARRT-AKI) trial was published and drew similar conclusions [9]. STARRT-AKI was a large multicentre study involving 168 ICUs across 15 countries and randomised 2927 patients with KDIGO stage 2 or 3 AKI (Table 1) into an accelerated RRT group (dialysis initiated within 12 h) and a standard group, where RRT was discouraged until AKI had persisted for more than 72 h or an urgent indication developed. Again, there was no significant difference in mortality (90 day mortality 43.9% accelerated vs 43.7% standard *p* = 0.92) [9].

Finally, ‘the comparison of two delayed strategies for renal replacement therapy initiation for severe acute kidney injury (AKIKI 2) study’ was published in 2021. It was a multicentre, prospective, open label, randomised controlled trial performed in 39 ICUs in France. Two-hundred and seventy-eight patients with oliguria for more than 72 h or a blood urea nitrogen concentration higher than 112 mg/dL (urea > 40 mmol/L) were randomised to receive RRT started just after randomisation or to a more-delayed strategy. With the more-delayed strategy, RRT initiation was postponed until a mandatory indication arose (hyperkalaemia, metabolic acidosis or pulmonary oedema) or when blood urea nitrogen concentration reached 140 mg/dL (urea 50 mmol/L). The primary outcome, the number of days alive and free of RRT between randomisation and day 28, was 12 days (IQR 0–25) vs 10 days (IQR 0–24) in the more-delayed strategy (*p* = 0.93). In a multivariable analysis, the hazard ratio for death at 60 days was 1.65 (95% CI 1.09–2.50, *p* = 0·018) with the more-delayed strategy.

Therefore, two trials examining the extremes of the time continuum, the ELAIN study examining the early end and the AKIKI-2 study examining the very late end, found harm associated with delay of RRT. By contrast, AKIKI, IDEAL-ICU and STARRT-AKI examined the middle of the spectrum (excluding patients who had early emergent indications but only delaying RRT for a limited period). Importantly, ELAIN compared early versus later administration of RRT rather than early versus expectant therapy, and the patient population was different to the other four studies. Many of the patients recruited into STARRT-AKI and the two AKIKI group studies had sepsis, and septic shock was part of the inclusion criteria for IDEAL-ICU, whereas most patients in ELAIN had a surgical diagnosis (93%) and close to half (47%) had undergone cardiac surgery. Furthermore, being a single centre study, the ELAIN trial may have inflated the treatment effects [14]. A comparable multicentre study investigating the timing of dialysis in cardiac surgery patients, the ‘High Volume Venovenous Hemofiltration Versus Standard Care for Post-Cardiac Surgery Shock’ (HEROICS) study, found no difference in mortality [15] and there was no significant difference in mortality among 965 surgical patients included in the STARRT-AKI study from early dialysis either (OR 0.96, 95% confidence interval 0.77–1.21) [9, 16].

Focusing on the evidence from the multicentre AKIKI, IDEAL-ICU and STARRT-AKI trials [7–9], we conclude that starting RRT early for a non-urgent indication does not reduce overall mortality. But does this mean timing of RRT is unimportant, or could there be a benefit in delaying RRT? The answer to this is clearer when the impact of timing on the proportion of patients who avoided dialysis is considered. In all three of these studies, 38–49% of patients avoided RRT in the expectant arm, compared to fewer than 10% in groups receiving early dialysis [7–9]. While this observation is undoubtedly a consequence of trial design, since patients receiving early dialysis did not have as much time to experience renal recovery, it is nonetheless an important one. Any patient who avoids RRT is spared the unnecessary costs and hazards associated with dialysis, such as thrombocytopenia, hypothermia, loss of micronutrients, hemodynamic instability and complications related to central venous access [3, 17]. Beyond individual patient care, avoiding unnecessary dialysis also represents responsible stewardship of finite healthcare resources.

However, clinicians should not simply interpret the findings of these studies as supportive of a delayed approach for everyone, but rather adopt a personalised approach where patients unlikely to require RRT are deferred, at least for a time, and patients likely to receive RRT are treated without delay. This personalisation was actually built in to the methodology of the STARRT-AKI trial; patients were excluded if the treating physicians had decided emergent RRT was necessary, or AKI recovery was imminent. Thus, only patients without an urgent indication for RRT that did not fit either category were randomised, resulting in the exclusion of over 7000 patients [9].

If we defer initiation of RRT in KDIGO stage 3 AKI patients without an urgent indication for RRT, how long can we safely delay therapy? AKIKI, IDEAL-ICU and STARRT-AKI compared early dialysis to a strategy of waiting up to 72 h [7–9]. What about patients who reach 72 h without developing an urgent indication? Do these patients benefit from an extended period of watchful waiting? The second trial from the AKIKI study group, AKIKI 2, would appear to provide the answer [10] (Table 2) since there was evidence of harm in the more-delayed arm [10]. Interestingly, these results were obtained from a study where the majority (nearly 80%) in the ‘more-delayed’ arm received RRT, much like the ELAIN study where 98% in the delayed arm received RRT [6]. Thus, consistent with the results of the ELAIN trial and earlier observational studies [5], we speculate that when RRT is ultimately administered, early is better.

Incorporating all these studies into clinical practice, we conclude that RRT can be safely deferred for up to 72 h in patients with KDIGO stage 3 AKI, provided the clinician is still uncertain as to whether RRT is inevitable, no life-threatening indication develops, and BUN remains < 112 mg/dL (Urea < 40 mmol/L). During this 72-h period of watchful waiting, management decisions should focus on enhancing the opportunity of AKI recovery. Cardiac output should be optimised, taking care to avoid excessive fluid resuscitation and hyperchloraemia. Venous congestion may decrease the pressure gradient across the glomerulus [18], while chloride-rich fluids may decrease renal perfusion [19, 20] and worsen acidosis [21]. Vasopressors should be used if mean arterial pressure (MAP) is consistently less than 65 mmHg [22, 23], and thoughtful consideration should be given to higher MAP targets (80 – 85 mmHg) in patients with chronic hypertension [24, 25], although recent evidence did not show improved renal outcomes with higher MAP targets in this cohort unless they were treated with angiotensin II receptor blockers prior to ICU admission [26]. More studies to inform, define and personalise ICU MAP targets are still required [27]. Drug choices should avoid classes frequently associated with nephrotoxic side effects [28] unless the therapeutic benefit outweighs the potential for harm, and drug dosing should consider the impact of impaired renal clearance [29], particularly for antibiotics [30, 31].

Despite these studies, critical care physicians are still called to exercise good clinical judgement when determining the best timing of RRT initiation in any given patient. Incipient renal failure cannot be seen in isolation and an assessment of organ dysfunction in other systems must be factored into decisions surrounding the initiation of RRT. For example, fluid overload will be less well tolerated in patients developing acute respiratory distress syndrome [32], as will metabolic acidosis contributing to ventilator dysynchrony [33]. When deferring RRT, clinicians should remain vigilant for the development of urgent indications. In the expectant arm of STARRT-AKI, over 60% of patients were commenced on RRT because of metabolic acidosis or hypoxemia due to fluid overload, perhaps partly explaining why the median time to dialysis was only 31 h, despite the protocol to wait for 72 h [9].

Regardless, it seems likely that some patients may be harmed by delaying RRT. In the AKIKI study, patients receiving RRT in the delayed group had a higher mortality than those in the early group (61.8% vs 48.5%, *p* < 0.001) [7], and, in the IDEAL-ICU study, 17% of the delayed group developed a requirement for urgent dialysis and these patients had higher mortality [8]. However, outcomes of patients receiving dialysis in delayed groups will likely be confounded by illness severity. Ultimately, better means to identify patients who will need dialysis earlier are urgently needed and approaches such as the furosemide stress test [34] or novel biomarkers [35] may be helpful for a more personalised approach to decision making.

## Conclusion

These studies give us confidence that when RRT is not yet urgently indicated and uncertainty remains whether AKI recovery might occur without RRT, expectant management may be practiced for a period of up to 72 h, while simultaneously ensuring conditions are optimised to provide the best chance of renal recovery. Despite the additional clarity provided by these studies, sound clinical judgement is still required to tailor RRT decisions for individual patients. As with so many other aspects of critical care, one size does not fit all.

## Data Availability

Not applicable.

## References

[1] Santos MJ, Martins MS, Santana FLP, Furtado MCSPC, Miname FCBR, Pimentel RRS (2020). COVID-19: instruments for the allocation of mechanical ventilators—a narrative review. Crit Care.

[2] Feinstein MM, Niforatos JD, Hyun I, Cunningham TV, Reynolds A, Brodie D (2020). Considerations for ventilator triage during the COVID-19 pandemic. Lancet Respir Med.

[3] Clark EG, Bagshaw SM (2015). Unnecessary renal replacement therapy for acute kidney injury is harmful for renal recovery. Semin Dial.

[4] Gaudry S, Quenot J-P, Hertig A, Barbar SD, Hajage D, Ricard J-D (2019). Timing of renal replacement therapy for severe acute kidney injury in critically Ill patients. Am J Resp Crit Care.

[5] Karvellas CJ, Farhat MR, Sajjad I, Mogensen SS, Leung AA, Wald R (2011). A comparison of early versus late initiation of renal replacement therapy in critically ill patients with acute kidney injury: a systematic review and meta-analysis. Crit Care.

[6] Zarbock A, Kellum JA, Schmidt C, Aken HV, Wempe C, Pavenstädt H (2016). Effect of early vs delayed initiation of renal replacement therapy on mortality in critically Ill patients with acute kidney injury: the ELAIN randomized clinical trial. JAMA.

[7] Gaudry S, Hajage D, Schortgen F, Martin-Lefevre L, Pons B, Boulet E (2016). Initiation strategies for renal-replacement therapy in the intensive care unit. N Engl J Med.

[8] Barbar SD, Clere-Jehl R, Bourredjem A, Hernu R, Montini F, Bruyère R (2018). Timing of renal-replacement therapy in patients with acute kidney injury and sepsis. N Engl J Med.

[9] The STARRT-AKI Investigators (2020). Timing of initiation of renal-replacement therapy in acute kidney injury. N Engl J Med.

[10] Gaudry S, Hajage D, Martin-Lefevre L, Lebbah S, Louis G, Moschietto S (2021). Comparison of two delayed strategies for renal replacement therapy initiation for severe acute kidney injury (AKIKI 2): a multicentre, open-label, randomised, controlled trial. Lancet.

[11] AKI definition (2012). KDIGO clinical practice guideline for acute kidney injury. Kidney Int Suppl.

[12] Meersch M, Küllmar M, Schmidt C, Gerss J, Weinhage T, Margraf A (2018). Long-term clinical outcomes after early initiation of RRT in critically Ill patients with AKI. J Am Soc Nephrol.

[13] Bellomo R, Ronco C, Kellum JA, Mehta RL, Palevsky P (2004). Acute renal failure—definition, outcome measures, animal models, fluid therapy and information technology needs: the Second International Consensus Conference of the Acute Dialysis Quality Initiative (ADQI) Group. Crit Care.

[14] Bellomo R, Warrillow SJ, Reade MC (2009). Why we should be wary of single-center trials. Crit Care Med.

[15] Combes A, Bréchot N, Amour J, Cozic N, Lebreton G, Guidon C (2015). Early high-volume hemofiltration versus standard care for post-cardiac surgery shock the HEROICS study. Am J Resp Crit Care.

[16] Magner K, Clark E, Hiremath S (2021). Letter to the editor regarding “Accelerated versus standard initiation of renal replacement therapy for critically ill patients with acute kidney injury: a systematic review and meta-analysis of RCT studies”. Crit Care.

[17] Douvris A, Zeid K, Hiremath S, Bagshaw SM, Wald R, Beaubien-Souligny W (2019). Mechanisms for hemodynamic instability related to renal replacement therapy: a narrative review. Intens Care Med.

[18] Legrand M, Dupuis C, Simon C, Gayat E, Mateo J, Lukaszewicz A-C (2013). Association between systemic hemodynamics and septic acute kidney injury in critically ill patients: a retrospective observational study. Crit Care.

[19] Chowdhury AH, Cox EF, Francis ST, Lobo DN (2012). A randomized, controlled, double-blind crossover study on the effects of 2-L infusions of 0.9% saline and plasma-Lyte® 148 on renal blood flow velocity and renal cortical tissue perfusion in healthy volunteers. Ann Surg.

[20] Reid F, Lobo DN, Williams RN, Rowlands BJ, Allison SP (2002). (Ab)normal saline and physiological Hartmann’s solution: a randomized double-blind crossover study. Clin Sci.

[21] Kellum JA (2002). Saline-induced hyperchloremic metabolic acidosis. Crit Care Med.

[22] Cecconi M, Backer DD, Antonelli M, Beale R, Bakker J, Hofer C (2014). Consensus on circulatory shock and hemodynamic monitoring. Task force of the European Society of Intensive Care Medicine. Intens Care Med.

[23] Rhodes A, Evans LE, Alhazzani W, Levy MM, Antonelli M, Ferrer R (2017). Surviving sepsis campaign: international guidelines for management of sepsis and septic shock: 2016. Intensive Care Med.

[24] Asfar P, Meziani F, Hamel J-F, Grelon F, Megarbane B, Anguel N (2014). High versus low blood-pressure target in patients with septic shock. N Engl J Med.

[25] Joannidis M, Druml W, Forni LG, Groeneveld ABJ, Honoré PM, Hoste E (2017). Prevention of acute kidney injury and protection of renal function in the intensive care unit: update 2017: expert opinion of the Working Group on Prevention, AKI section, European Society of Intensive Care Medicine. Intensive Care Med.

[26] Demiselle J, Seegers V, Lemerle M, Meziani F, Grelon F, Megarbane B (2021). Prior exposure to angiotensin II receptor blockers in patients with septic shock to individualize mean arterial pressure target? A post hoc analysis of the sepsis and mean arterial pressure (SEPSISPAM) trial*. Crit Care Med.

[27] Jörres A (2021). Initial arterial blood pressure targets in patients with septic shock: one size fits all or made to measure?*. Crit Care Med.

[28] Pierson-Marchandise M, Gras V, Moragny J, Micallef J, Gaboriau L, Picard S (2017). The drugs that mostly frequently induce acute kidney injury: a case—noncase study of a pharmacovigilance database. Brit J Clin Pharmaco.

[29] Dowling TC, Matzke GR, Murphy JE, Burckart GJ (2010). Evaluation of renal drug dosing: prescribing information and clinical pharmacist approaches. Pharmacother J Hum Pharmacol Drug Ther.

[30] Eyler RF, Mueller BA (2011). Antibiotic dosing in critically ill patients with acute kidney injury. Nat Rev Nephrol.

[31] Lewis SJ, Mueller BA (2014). Antibiotic dosing in patients with acute kidney injury. J Intensive Care Med.

[32] Wiedemann HP, Wheeler AP, Bernard GR, Thompson BT, Hayden D, Network NH Lung, and Blood Institute Acute Respiratory Distress Syndrome (ARDS) Clinical Trials (2006). Comparison of two fluid-management strategies in acute lung injury. N Engl J Med.

[33] Blanch L, Villagra A, Sales B, Montanya J, Lucangelo U, Luján M (2015). Asynchronies during mechanical ventilation are associated with mortality. Intensive Care Med.

[34] Lumlertgul N, Peerapornratana S, Trakarnvanich T, Pongsittisak W, Surasit K, Chuasuwan A (2018). Early versus standard initiation of renal replacement therapy in furosemide stress test non-responsive acute kidney injury patients (the FST trial). Crit Care.

[35] Hoste E, Bihorac A, Al-Khafaji A, Ortega LM, Ostermann M, Haase M (2020). Identification and validation of biomarkers of persistent acute kidney injury: the RUBY study. Intens Care Med.

